# Evaluating smoking cessation interventions in China’s primary care facility networks for hypertensive patients aged 45 years and older: A retrospective cohort study

**DOI:** 10.18332/tid/169975

**Published:** 2023-09-27

**Authors:** Yang Wang, Ludwing F. Salamanca, Carmen S. Sanchez, Hai Fang

**Affiliations:** 1 School of Public Health, Peking University, Beijing, China; 2 China Center for Health Development Studies, Peking University, Beijing, China; 3 Department of Psychiatry, Columbia University, New York, United States; 4 New York State Psychiatric Institute, New York, United States; 5 Department of Preventive Medicine and Public Health, Food Sciences, Toxicology and Legal Medicine, Faculty of Medicine, University of Valencia, Valencia, Spain

**Keywords:** smoking, tobacco cessation, aging, primary care, public health

## Abstract

**INTRODUCTION:**

In China’s aging population, hypertension, aggravated by smoking, constitutes a substantial health concern. However, the effectiveness of the national public health interventions for smoking cessation under the Essential Public Health Services remains unverified. Our aim was to examine smoking prevalence, the proportion of individuals receiving smoking cessation advice, its impact on successful cessation among Chinese hypertensive patients aged ≥45 years, and to discern disparities in the coverage and efficacy of smoking cessation interventions between primary care facilities and hospitals.

**METHODS:**

Employing a longitudinal cohort approach, we examined four waves (2011–2018) of the China Health and Retirement Longitudinal Study (CHARLS). We surveyed a nationally representative cohort of hypertensive individuals, accounting for smoking status, cessation advice, healthcare preferences, and 11 pertinent covariates.

**RESULTS:**

Among the 4269 hypertensive patients, smokers were predominantly male, aged <65 years, with lower education and lower wealth. Smoking prevalence decreased from 25.2% in 2011 to 21.1% in 2018. The incidence of physician-provided smoking cessation advice reached a peak of 60.3% in 2015, decreasing to 54.8% in 2018. Visitors to primary care facilities reported the highest rate of advice reception. While patients receiving advice exhibited a higher smoking prevalence, instrumental variable regression and subgroup analyses found no significant correlation between advice receipt and successful cessation.

**CONCLUSIONS:**

We observed a substantial smoking prevalence among older hypertensive Chinese individuals and limited effectiveness of existing cessation programs. This underscores the necessity for augmenting primary care and devising a comprehensive health policy for more successful smoking cessation interventions, particularly considering China’s rapidly aging population.

## INTRODUCTION

Hypertension can lead to several severe complications such as cardiovascular diseases, cerebrovascular events, and kidney damage, which may precipitate premature death^1,2^. Aging is a significant contributing factor to hypertension; prevalence often exceeds 50% among individuals aged ≥60 years, a rate that is markedly 20% higher than in the general adult population^3^. Smoking is another key risk factor for hypertension. Although direct evidence for smoking as a hypertension cause is not conclusive^4^, consistent findings suggest that it significantly worsens the prognosis for hypertensive patients^5^. Moreover, elderly smokers tend to have higher all-cause mortality and an increased likelihood of physical and cognitive impairments compared to non-smokers^6^. A large-scale study revealed that the coexistence of hypertension, smoking, and aging results in a risk of cardiovascular disease that is over 50% higher for smokers aged >60 years with hypertension than for non-smokers^7^.

The concurrent prevalence of hypertension, smoking, and aging significantly contributes to the chronic, progressive decline of population health. Given this, it is vital to integrate smoking cessation interventions specifically for elderly smokers into accessible, equitable, and continuous health services. Compared to secondary care, primary care offers considerable advantages in providing these services, especially to at-risk populations. These services include early prevention, continuity, and person-centered care^8^, which align seamlessly with the holistic approach of the multicomponent smoking cessation treatment^9^. This treatment encourages physicians to take into account a multitude of factors, encompassing the biological, psychological, and social facets of smokers. It also offers an extended series of comprehensive interventions aimed at facilitating smoking cessation^10^. Therefore, primary care is commonly regarded as the most suitable setting for administering smoking cessation treatment^11^.

Since 2009, China has initiated a secondary preventive smoking cessation intervention program targeting hypertensive patients aged ≥35 years^12^. This program, embedded within the Essential Public Health Services (EPHS) program for hypertensive patients, is supported by government funding and aims to encompass community residents. In practice, the intervention is primarily delivered by family doctor teams or primary care practitioners (PCPs) within China’s primary care facility network. They recruit eligible hypertensive patients from local communities who voluntarily sign up to receive quarterly consultations each year^13^. According to the guidelines, family doctor teams or PCPs are required to inquire about the smoking habits of the target patients during each visit. They provide health education to help patients understand the risks of smoking, encourage them to reduce or quit smoking, and prevent relapse by offering lifestyle change advice. They also recommend the use of nicotine patches or bupropion for patients experiencing significant withdrawal symptoms^13,14^. However, the 2009 national guidelines provided relatively vague instructions regarding the details of psychoeducation, behavior change, treatment duration, and medication standards^14^. This lack of specificity may result in the intervention pathway being more dependent on the individual experiences and discretion of the practitioners.

Five years after the initiation of this program, surveys indicated a smoking rate of 25.6% among the elderly population^15^. This is particularly concerning given the high prevalence of hypertension, which stood at 58.4% in 2018 among the same demographic^16^. Simultaneously, a nationwide study revealed a disconcerting statistic: the effective control rate of hypertension among Chinese patients was only 15.3%^3^. This low rate suggests potential limitations in the coverage and effectiveness of interventions provided by the Essential Public Health Services (EPHS), which include smoking cessation. Given these findings, and considering the vague stipulations in the guidelines, there are growing concerns about the actual effectiveness of the smoking cessation component of the program and underscore the urgent need to evaluate it.

In this study, our objective was to carry out a retrospective cohort analysis to estimate the smoking prevalence among hypertensive patients aged ≥45 years in China, and to identify the proportion of these hypertensive smokers who received smoking cessation advice from physicians between 2011 and 2018. We also analyzed the correlation between receiving such advice and the subsequent cessation of smoking among these individuals. Moreover, we investigated the prevalence and efficacy of receiving smoking cessation advice among patients who predominantly visit primary care facilities, in comparison to those who primarily attend hospitals, to discern whether patients in primary care settings are more likely to receive preventive medical interventions in China.

## METHODS

### Data sources and study population

We conducted a population-based longitudinal cohort analysis using data from four waves (2011, 2013, 2015, and 2018) of the China Health and Retirement Longitudinal Study (CHARLS)^17^. This nationally representative cohort collects high-quality data from individuals aged ≥45 years residing in China through face-to-face interviews conducted by trained interviewers from Peking University. The participant selection process encompassed 450 communities and villages, employing a multi-stage, proportional sampling method. Further information on CHARLS can be found in other publications^18^.

In 2011, CHARLS interviewers assessed blood pressure for surveyed residents using the OmronTM HEM-7200 Monitor, and residents were also queried about hypertension status. Our study population included individuals diagnosed with hypertension (systolic/diastolic blood pressure higher than 140/90 mmHg) in 2011, as defined by the Guidelines for the Prevention and Treatment of Hypertension in China^14^.

### Smoking status and smoking cessation advice

During the 2011–2018 surveys, residents were inquired about their smoking status: ‘Do you still have the habit of smoking or have you completely quit?’. We determined participant smoking status based on their responses. In the four CHARLS survey waves from 2011 to 2018, participants reporting hypertension were asked a series of questions related to EPHS. One question was: ‘In the past year (last 12 months), have your care providers given your health education/advice on smoking cessation?’. In the smoking control inquiry, patients could respond with ‘yes’ or ‘no’. Based on this response, we determined whether hypertensive smokers had received smoking cessation advice as part of EPHS in the previous year.

### Frequent visitors to primary care facilities and hospitals

In China, healthcare institutions are classified into two categories: hospitals and primary care facilities. The latter primarily focus on providing EPHS and primary care. In CHARLS, each respondent was asked two questions across all four waves: ‘Which types of medical facilities have you visited in the last four weeks for outpatient treatment?’ and ‘How many times did you visit (this type of facility) during the last month?’. Based on these two questions, we selected residents who visited primary care facilities at least twice and never visited a hospital, and those who visited a hospital at least twice and never visited primary care facilities in the four waves to represent residents who frequently sought healthcare at primary care facilities or hospitals. Additionally, we selected a group of residents who did not report visiting any healthcare facilities in all four surveys as a subgroup with limited healthcare use, to be compared with the two subgroups of interest.

### Covariates

Aligned with the Chinese Hypertension Clinical Guidelines and previous study on smoking cessation outcomes in China^14,18^, we incorporated 11 variables as covariates. These include gender, age, marital status, region (divided into eastern and non-eastern regions), living area (urban/rural), education level, wealth (lowest 25%, 26%–50%, 51%–75%, and highest 25%), presence or absence of Activities of Daily Living (ADL) disability, Instrumental Activities of Daily Living (IADL) disability, and presence of complications attributable to hypertension: cardiovascular disease, stroke, and kidney disease. We followed existing literature for definitions of ADL and IADL disabilities^19^. For international comparison, age was divided into 45–54, 55–64, 65–74, and ≥75 years^20,21^. We split the regions into eastern and non-eastern due to documented healthcare inequity between the economically developed eastern China and other regions^22^.

### Statistical analysis

We conducted descriptive statistical analyses on the sociodemographic characteristics of hypertensive patients who smoked or did not smoke in 2011, changes in smoking prevalence among hypertensive patients from 2011 to 2018, and changes in the proportion of smokers receiving smoking cessation advice. Age adjustment was performed using direct standardization with the standard being all adults with hypertension from 2012 to 2015; the age categories used for standardization were 45–54 (30.8%), 55–64 (31.5%), 65–74 (23.1%), and ≥75 years (14.4%)^23^. We also examined the proportion of smokers receiving advice in three subgroups: frequent visitors to primary care facilities, hospitals, and those with limited healthcare use.

After collinearity tests, we built a generalized estimating equation model with obtained smoking cessation advice in the last 12 month as the independent variable and smoking status as the outcome. We included all covariates potentially associated with smoking prevalence (p≤0.2) and used a stepwise backward deletion approach to remove non-significant variables.

For sensitivity analysis, we employed two instrumental variables for longitudinal regression. This was due to the potential risk of reverse causality between smoking status and receiving smoking cessation advice: local PCPs might have provided more smoking cessation interventions due to the patients’ smoking habits^24^. To minimize threats to internal validity, we selected two instrumental variables from the CHARLS dataset that had no direct association with smoking: the receipt of exercise advice and weight loss advice from doctors in the past 12 months. As part of the EPHS lifestyle interventions for hypertensive patients, the receipt of exercise and weight loss advice could partially indicate whether patients used these services. However, the primary target population for these services was overweight or obese patients, with no direct relationship to smoking status. After conducting endogeneity, weak instrumental variable, and over identification tests, we further examined the causal relationship.

Lastly, we ran similar models within two subgroups, primary care facility visitors and hospital visitors, to investigate any association between receiving smoking cessation advice and smoking cessation outcomes. All analyses were performed using Stata 17.0. Tests were two-tailed, with p≤0.05 as the significance level.

## RESULTS

Our study encompassed 4269 hypertensive patients, of which 36.5% were aged ≥65 years and 44.3% were male (Table 1). Among the smokers in this cohort, we found a significantly larger proportion of males (86.8% vs 30.4%, p≤0.001), those aged <65 years (68.1% vs 62.0%, p≤0.001), those with an education level of primary school or lower (42.8% vs 38.0%, p≤0.001), those in the lowest 25% wealth bracket (29.3% vs 23.8%, p≤0.001), and those without an IADL disability (79.6% vs 69.5%, p≤0.001), compared to non-smokers.

**Table 1:** Sociodemographic characteristics of hypertensive patients who were currently smoking or not smoking in 2011 (N=4269)

*Characteristics*	*Not smoking*		*Smoking*		*Total*		*p*
	*n*	*%*	*n*	*%*	*n*	*%*
**Total**	3220	100	1049	100	4269	100	
**Age** (years)							≤0.001
45–54	759	23.57	281	26.79	1040	24.36	
55–64	1237	38.42	433	41.28	1670	39.12	
65–74	805	25.00	243	23.16	1048	24.55	
≥75	419	13.01	92	8.77	511	11.97	
**Gender**							≤0.001
Male	980	30.43	910	86.75	1890	44.27	
Female	2240	69.57	139	13.25	2379	55.73	
**Marital status**							0.011
Married	2525	78.42	861	82.08	3386	79.32	
Not married	695	21.58	188	17.92	883	20.68	
**Education level**							≤0.001
No formal education	1073	33.32	189	18.02	1262	29.56	
Primary school or lower	1222	37.95	449	42.80	1671	39.14	
Middle school	533	16.55	277	26.41	810	18.97	
High school or higher	392	12.17	134	12.77	526	12.32	
**Region**							0.713
Eastern region	1076	36.07	345	35.42	1421	35.91	
Non-eastern region	1907	63.93	629	64.58	2536	64.09	
**Residence**							0.018
Urban	1491	46.30	442	42.14	1933	45.28	
Rural	1729	53.70	607	57.86	2336	54.72	
**Wealth (%)**							≤0.001
Lowest 25	766	23.79	307	29.27	1073	25.13	
26–50	814	25.28	254	24.21	1068	25.02	
51–75	796	24.72	277	26.41	1073	25.13	
Highest 25	844	26.21	211	20.11	1055	24.71	
**Presence of ADL disability**							0.35
Yes	985	30.59	337	32.13	1322	30.97	
No	2235	69.41	712	67.87	2947	69.03	
**Presence of IADL disability**							≤0.001
Yes	983	30.53	214	20.40	1197	28.04	
No	2237	69.47	835	79.60	3072	71.96	
**Heart disease**							0.001
Yes	832	25.84	218	20.78	1050	24.60	
No	2388	74.16	831	79.22	3219	75.40	
**Stroke**							0.051
Yes	231	7.17	57	5.43	288	6.75	
No	2989	92.83	992	94.57	3981	93.25	
**Kidney disease**							0.51
Yes	232	7.20	82	7.82	314	7.36	
No	2988	92.80	967	92.18	3955	92.64	

**Figure 1:**
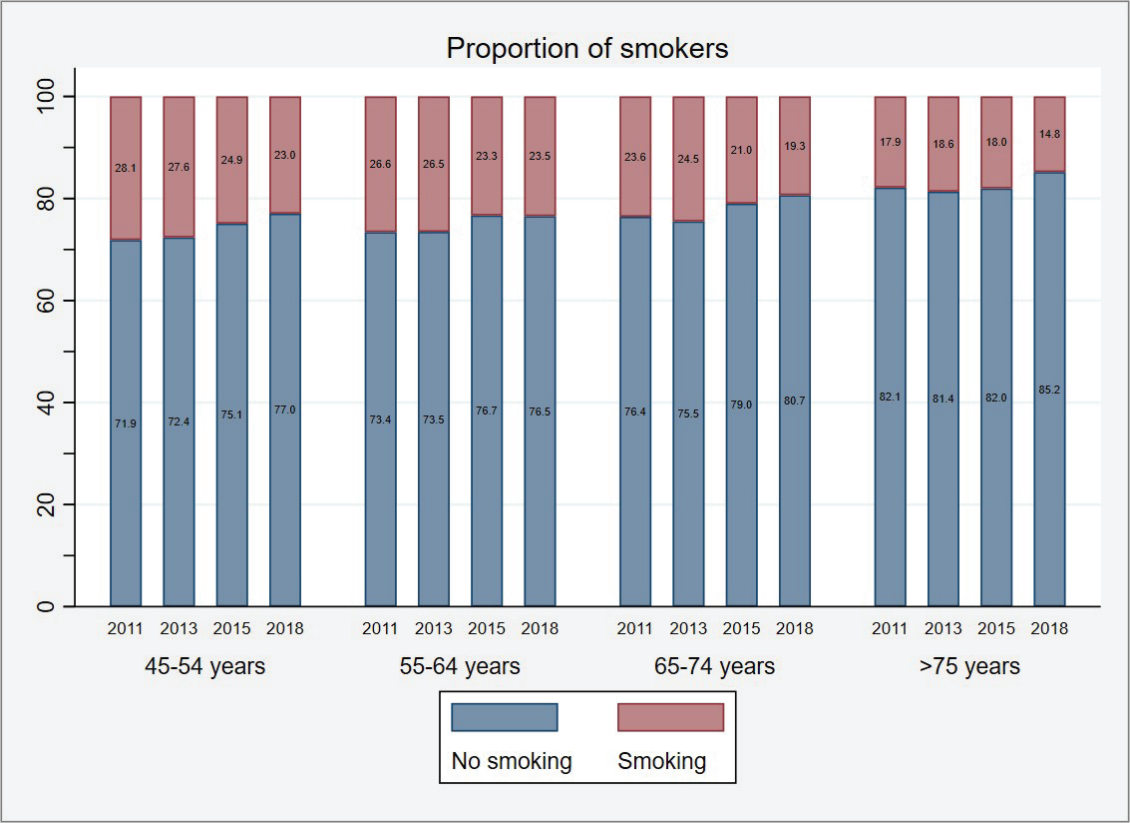
The smoking prevalence among hypertensive patients from 2011 to 2018

After adjusting for age, the smoking prevalence among hypertensive patients surveyed in 2011 was 25.1%. This rate remained stable at 25.2% in 2013 but began to decrease in 2015 to 22.5% and further declined to 21.1% by 2018. An inverse relationship was observed between the age of hypertensive patients and their smoking prevalence. In 2011, for instance, patients aged ≥75 years had a smoking prevalence that was 10.2% lower than that of patients aged 45–54 years. Furthermore, all age groups demonstrated a consistent decline in smoking prevalence over time. Between 2011 and 2018, the smoking prevalence decreased by 5.1%, 3.1%, 4.3%, and 3.1% for the age groups of 45–54, 55–64, 65–74, and ≥75 years, respectively (Figure 1).

**Figure 2:**
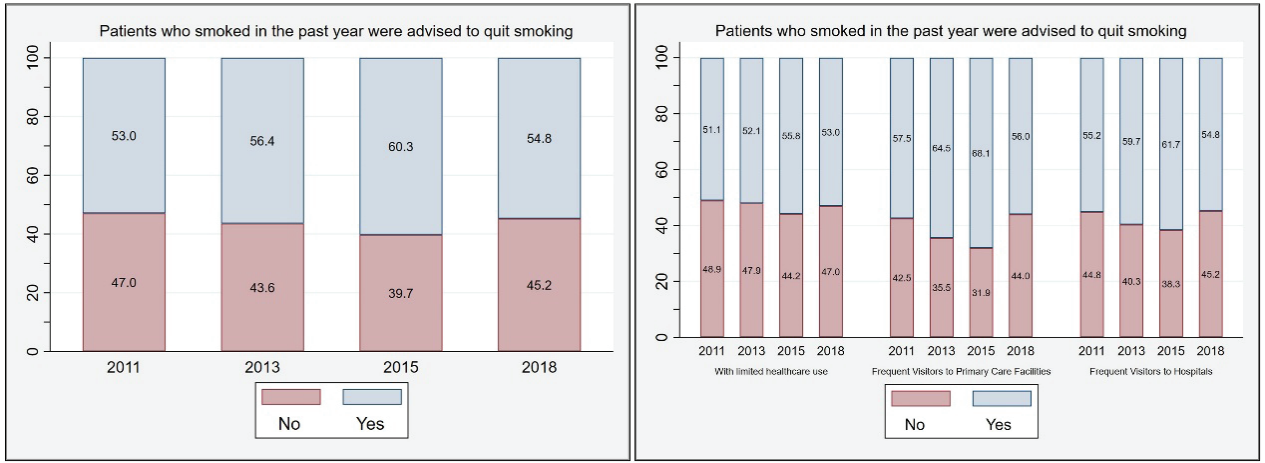
The proportion of smokers who were advised by doctors to quit smoking in the past year between 2011 and 2018.

As presented in Figure 2, in 2011, only 53% of hypertensive patients who smoked reported having received smoking cessation advice from their physicians. This figure rose to a peak of 60.3% in 2015, before declining to 54.8% in 2018. Patients with frequent visits to primary care facilities had the highest rates of receiving smoking cessation advice, peaking at 68.1% in 2015. This group also showed a consistently higher percentage of receiving such advice, with a range of 1.2–6.4% more than those who frequently visited hospitals. Similarly, patients with restricted healthcare access showed a 3.0–12.3% lower rate compared to frequent primary care facility visitors.

**Figure 3:**
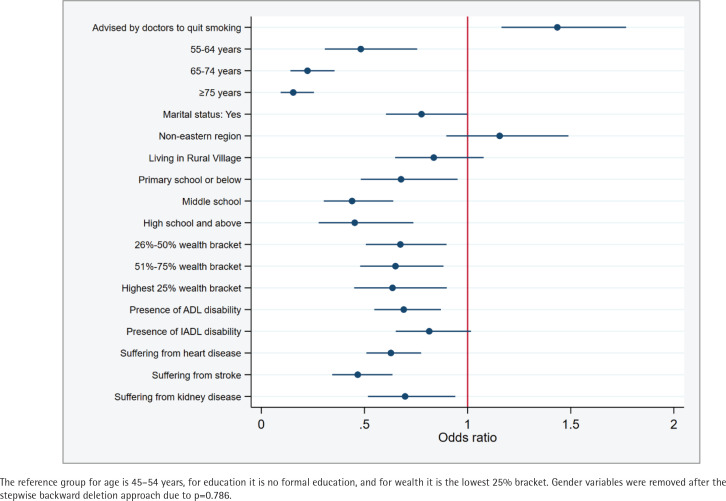
Generalized estimating equation: association between receiving smoking cessation advice from doctors and smoking

**Figure 4:**
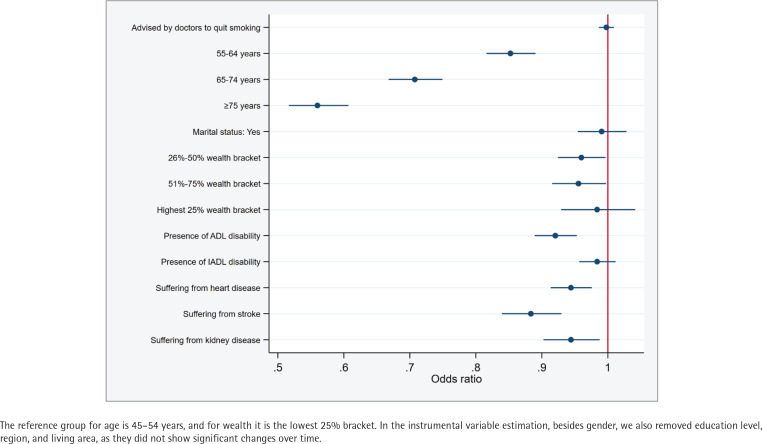
Instrumental variable estimation: association between receiving smoking cessation advice from doctors and smoking

Findings from the generalized estimating equation suggested that hypertensive patients who received smoking cessation advice exhibited a 1.435 times higher prevalence of smoking (95% CI: 1.16–1.77, p=0.001) than those who did not receive such advice (Figure 3). However, this ratio was reduced to 0.998 in the instrumental variable regression, thus nullifying its significant association (95% CI: 0.99– 1.01, p=0.694) (Figure 4). Moreover, no substantial association was found between the receipt of smoking cessation advice and successful smoking cessation among hypertensive patients who frequently visited primary care facilities and hospitals (Supplementary file Table S1).

## DISCUSSION

Our study presents three primary findings. Firstly, in 2011, nearly 25% of Chinese hypertensive adults aged >45 years were smokers, with the rate decreasing to around one fifth by 2018. Secondly, at most between 50% and 60% of these smoking patients reported receiving doctors’ advice on smoking cessation during these seven years, with individuals who frequently visited primary care facilities being more likely to obtain such advice than those who frequently visited hospitals. Lastly, it appears that the advice did not significantly reduce the smoking prevalence in this population during this period.

While chronic diseases serve as a catalyst for older adults to quit smoking^25^, and despite a decrease in smoking rates among hypertensive older adults in China over a seven-year period, our study determined that the prevalence of smoking in this demographic was not markedly different from that of the overall elderly population^15^. Although this rate was lower than those observed in populous, low-to-middle-income Asian countries like India and Indonesia (both exceeding 40%)^26,27^, it was significantly higher than the rates in the general populations of the United States and Europe for comparable age groups and years. Specifically, these rates were 1.4, 1.9, and 3.3 times higher for the age groups of 55–64, 65–74, and ≥75 years, respectively, compared to the US^20^, and 1.8 and 2.2 times higher for the age groups of 65–74 and ≥75 years in Europe^21^. Given China’s rapidly aging population, these consistently high smoking rates present a significant epidemiological challenge, underlining the urgent need for effective and equitable smoking cessation interventions and treatments within the healthcare system.

China’s existing healthcare system, dominated by secondary care, follows a disease-centered, profit-driven model^28^. Secondary care, constituted mainly by general hospitals situated primarily in the eastern regions, is known for its high fees. Given China’s lower per capita income and a 30% average healthcare insurance co-payment ratio, these hospitals pose a challenge to achieving equitable healthcare^22,29^. In contrast, the primary care system, encompassing approximately a million facilities scattered across urban and rural areas, offers improved accessibility and promotes health equity^22^. Previous research has indicated that primary care is predominantly utilized by rural residents, individuals with lower wealth, and those residing in less economically developed regions^30^. Coincidentally, our study observed a higher proportion of vulnerable populations among smokers. We further discovered that primary care facilities in China are more likely to provide smoking cessation advice than general hospitals. These findings underscore the urgent need to bolster primary care, specifically in enhancing its accessibility and equity in delivering preventive services, such as smoking cessation interventions.

Our study also discovered the potential limitations in the coverage and effectiveness of China’s current primary care-led smoking cessation intervention. These issues may be rooted in the trade-offs inherent in EPHS health policy, which seems to have prioritized low cost over other factors. From 2011–2018, the government’s annual per capita expenditure for EPHS was only 40 Chinese Renminbi (about US$7)^13^. This financial constraint could have impacted the overall performance of EPHS in terms of equity and health^31^. Our study reveals that between 2011 and 2018, only 50% to 60% of hypertensive smokers were provided with smoking cessation advice. Notably, in 2015, the percentage of recipients peaked at 60.3%, potentially spurred by a significant policy report that year which underscored the dangers of smoking to the health of China’s population^32^. However, by 2018, this proportion had regrettably diminished. Besides, from a clinical intervention viewpoint, current strategies appear to fall short in several areas: ambiguous guidelines for EPHS smoking cessation interventions and hypertensive patient management^14^; cessation rates are not included in EPHS performance metrics^12^; family doctor teams and public health practitioners might lack adequate knowledge and skills for delivering smoking cessation treatments^33^; and resources for referring smokers to specialized clinics are insufficient. It is worth emphasizing that treatments and medications for smoking cessation for hypertensive patients are not covered by China’s medical insurance payments^34^. This results in doctors potentially giving largely discretionary verbal recommendations about taking smoking cessation medications, with patients then having to purchase these medications over the counter. According to a 2018 national survey of adults, of smokers aged ≥45 years attempting to quit, fewer than 3% utilized smoking cessation medications^35^. Collectively, these issues may compromise the effectiveness of smoking cessation interventions within the current EPHS framework.

Currently, a wealth of clinical evidence confirms that both brief and intensive clinical interventions can effectively assist individuals in quitting smoking^36^, and the implementation of corresponding health policies at the national level can further improve public health^37^. For instance, in the United Kingdom, nationwide smoking cessation services are administered through the National Health Service’s primary care trusts. These trusts primarily deliver smoking cessation services via qualified counselors, who are eligible for funding reimbursement^38^. In the United States, major medical insurance covers evidence-based smoking cessation treatments and medication reimbursement, facilitating easier access to services from clinical physicians for smokers^39^. In Spain, smoking cessation services and medication reimbursement are integrated into primary care and other health services through public health and preventive medicine programs. These programs are supported by local health departments and primary care association^40^. Regardless of the divergences in health system models, national health financial commitments, and the maturity of primary care, these countries have shared experiences that may prove beneficial for China’s consideration: future effective population-based smoking cessation interventions necessitate a comprehensive system design that spans from health policy to clinical intervention, and encapsulates several key components such as health financing, health service provision, performance measurement, clinical guideline development, pharmaceutical resources, and personnel training.

### Limitations

Our study has several limitations. Firstly, our findings are primarily relevant to China and may not be directly generalizable to other countries with different healthcare systems and cultural norms. Secondly, the CHARLS survey, as a periodic panel cohort survey, inherently limits our ability to track the onset and progression of hypertension in real-time. This design only allows us to determine whether a respondent had hypertension at the specific time of each survey iteration, which may lack precision. As a result, we did not incorporate the duration of hypertension into our analysis. Thirdly, the data on whether patients received smoking cessation advice were gathered from patients’ recollections, potentially leading to recall bias. Lastly, the patients’ smoking status relied on self-reported data rather than objective measurements, which may create discrepancies between actual and reported smoking status. Despite these limitations, we believe the large sample size in our study and robust methodological approach helps to mitigate these biases.

## CONCLUSIONS

The study found high smoking rates among hypertensive Chinese adults aged >45 years, with smoking cessation advice from physicians failing to significantly decrease this prevalence between 2011 and 2018. Notably, primary care facilities, not general hospitals, were more likely to provide such advice. These findings highlight the urgency of enhancing primary care and formulating a comprehensive health policy for more effective delivery of smoking cessation interventions, given China’s rapidly aging population.

## Data Availability

The data supporting this research are available from the authors on reasonable request.
