# Supplementary material for: Evaluating smoking cessation interventions in China’s primary care facility networks for hypertensive patients aged 45 years and older: A retrospective cohort study
Source: Tob Induc Dis. 2023 Sep 27;21:119. doi: 10.18332/tid/169975 (PMC10523951; doi:10.18332/tid/169975)
Supplement: Supplementary file 1 [file TID-21-119-s1.pdf]

Table S1: Generalized Estimating Equation: Association between receiving smoking cessation advice from doctors and smoking for patients who frequently visited primary care facilities or hospitals

	Model 1 Frequent visitors to primary care facilities			Model 2 Frequent visitors to hospitals		
	OR	95%CI	P value	OR	95%CI	P value
**Advised by doctors to quit smoking**						
**No**	1	[1.00,1.00]		1	[1.00,1.00]	
**Yes**	1.529	[0.89,2.64]	0.127	1.95	[0.95,4.00]	0.069
**Age**						
**45-54 years**	1	[1.00,1.00]		1	[1.00,1.00]	
**55-64 years**	0.342	[0.11,1.11]	0.074	0.258	[0.05,1.26]	0.094
**65-74 years**	0.133**	[0.04,0.48]	0.002	0.117**	[0.02,0.55]	0.007
**≥75 years**	0.0541***	[0.01,0.21]	<0.01	0.0937**	[0.02,0.57]	0.01
**Education**						
**No formal education**	1	[1.00,1.00]				
**Primary school or below**	0.693	[0.33,1.46]	0.334			
**Middle school**	0.287**	[0.13,0.66]	0.003			
**High school and above**	0.265*	[0.10,0.73]	0.01			
**Wealth**						
**Lowest 25%**	1	[1.00,1.00]		1	[1.00,1.00]	
**26%-50%**	0.564	[0.29,1.08]	0.084	0.907	[0.30,2.74]	0.863
**51%-75%**	0.791	[0.36,1.76]	0.565	0.495	[0.16,1.52]	0.221
**Highest 25%**	0.768	[0.32,1.82]	0.548	0.358	[0.09,1.35]	0.13
**Presence of ADL disability**						
**No**				1	[1.00,1.00]	
**Yes**				0.339**	[0.16,0.71]	0.004
**Presence of IADL disability**						
**No**				1	[1.00,1.00]	
**Yes**				0.453*	[0.24,0.85]	0.014
**Suffering from heart disease**						
**No**	1	[1.00,1.00]				
**Yes**	0.570*	[0.34,0.96]	0.034			
**Suffering from stroke**						
**No**	1	[1.00,1.00]				
**Yes**	0.424*	[0.19,0.94]	0.036			
**N**		637			256	

Note:

1. * $p < 0.05$, ** $p < 0.01$, *** $p < 0.001$

2. In Model 1, we removed the following variables after the stepwise backward deletion approach: Marital status (p=0.773), Region (P=0.891), Living area (p=0.666), Presence of ADL disability (p=0.269), Presence of IADL disability (p=0.209), and Suffering from kidney disease (p=0.532).

3.In Model 2, we excluded the following variables after the stepwise backward deletion approach: Marital status (p=0.397), Education

(p=0.353-0.558), Region (P=0.548), Living area (p=0.438), Suffering from heart disease (p=0.484), Suffering from stroke (p=0.966), and Suffering from kidney disease (p=0.318).

©2023 Wang Y. et al.
